# Effects of *Bacillus coagulans* TBC169 on gut microbiota and metabolites in gynecological laparoscopy patients

**DOI:** 10.3389/fmicb.2024.1284402

**Published:** 2024-03-26

**Authors:** Weiqi Gao, Ya Yan, Zhaobo Guan, Jingmin Zhang, Weihong Chen

**Affiliations:** ^1^Third Hospital of Shanxi Medical University, Shanxi Bethune Hospital, Shanxi Academy of Medical Sciences, Taiyuan, China; ^2^Tongji Hospital, Tongji Medical College, Huazhong University of Science and Technology, Wuhan, China; ^3^School of Pharmacy, Shanxi Medical University, Taiyuan, China

**Keywords:** *Bacillus coagulans*, gynecological laparoscopic surgery, intestinal function, gut microbiota, metabolite

## Abstract

**Objective:**

The primary objective of this study is to investigate the mechanism by which *Bacillus coagulans* TBC169 accelerates intestinal function recovery in patients who have undergone gynecological laparoscopic surgery, using metabolomics and gut microbiota analysis.

**Methods:**

A total of 20 subjects were selected and randomly divided into two groups: the intervention group (*n* = 10) receiving *Bacillus coagulans* TBC169 Tablets (6 pills, 1.05 × 10^8^ CFU), and the control group (*n* = 10) receiving placebos (6 pills). After the initial postoperative defecation, fecal samples were collected from each subject to analyze their gut microbiota and metabolic profiles by high-throughput *16S* rRNA gene sequencing analysis and untargeted metabonomic.

**Results:**

There were no statistically significant differences observed in the α-diversity and β-diversity between the two groups; however, in the intervention group, there was a significant reduction in the relative abundance of *unclassified_Enterobacteriaceae* at the genus level. Furthermore, the control group showed increased levels of *Holdemanella* and *Enterobacter*, whereas the intervention group exhibited elevated levels of *Intestinimonas*. And administration of *Bacillus coagulans* TBC169 led to variations in 2 metabolic pathways: D-glutamine and D-glutamate metabolism, and arginine biosynthesis.

**Conclusion:**

This study demonstrated that consuming *Bacillus coagulans* TBC169 after gynecological laparoscopic surgery might inhibit the proliferation of harmful *Enterobacteriaceae*; mainly influence 2 pathways including D-glutamine and D-glutamate metabolism, and arginine biosynthesis; and regulate metabolites related to immunity and intestinal motility; which can help regulate immune function, maintain intestinal balance, promote intestinal peristalsis, and thus accelerate the recovery of intestinal function.

## 1 Introduction

Laparoscopy is a widely employed technique in the field of gynecology, offering several advantages compared to traditional surgery, such as reduced postoperative pain, decreased risk of complications, shorten hospitalization time, enhanced postoperative recovery, (Lee et al., 2010). The rapid recovery of intestinal motility after gynecological laparoscopies (GLs) is crucial, as it serves as a predictor for favorable postoperative clinical outcomes (Greenwood-Van et al., 2017). Conversely, delayed recovery of intestinal motility can impede postoperative recovery progress, resulting in increased gastrointestinal symptoms and higher treatment costs (Wu et al., 2022).

Several approaches have been utilized to facilitate the recovery of intestinal motility, such as chewing gum, low-frequency electrical stimulation, coffee consumption, et al., although no consensus has been reached on this issue at present. Some of these methods are still unclear in effect; some are complex in operation; and some need to be combined with other enhanced recovery methods (Gungorduk et al., 2020; Turkay and Yavuz, 2020; Wu et al., 2022). Modifying microbiota-gut-brain interactions through probiotics has emerged as a promising therapeutic approach for gut motility disorders (Tillisch et al., 2013). *Bacillus coagulans* belongs to spore-forming organisms, which could promote the peristaltic activity of intestine, strengthen the health of intestinal cells and improve the intestinal microenvironment (Ara et al., 2002; Minamida et al., 2015). A recent study revealed the effective promotion of intestinal function recovery in patients post-GLs using *Bacillus coagulans* TBC169 (1.05 × 10^8^ CFU, 3 times daily), with no reported adverse events (Li et al., 2022). Despite the effect of *Bacillus coagulans* TBC169 was evident, its underlying mechanism had not been investigated.

## 2 Materials and methods

### 2.1 Study design

**Figure 1:**
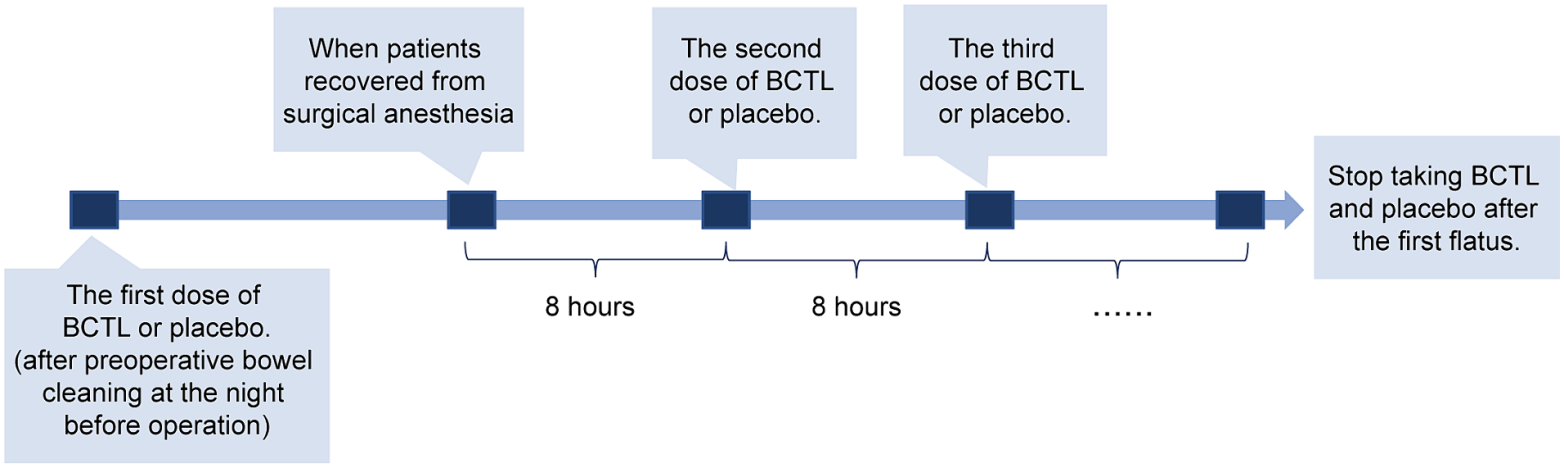
Schematic representation of patient medication administration. Patients took the first dose of BCTL or placebo after preoperative bowel cleaning at the night before operation; and since the patients woke up from anesthesia, they were administered BCTL or placebo every 8 h until the first flatus after surgery.

This study enrolled 20 subjects who underwent laparoscopic surgery in the gynecology department of Shanxi Bethune Hospital between July and December 2022. The inclusion criteria were as follows: individuals who were aged between 30 and 70 years, underwent GLs, and the operation duration ranging from 1 to 4 h. The exclusion criteria included psychiatric disorders, a history of intestinal operation, drug allergy to probiotics, and poor compliance. Finally 20 patients who met the inclusion criteria were selected and then randomly assigned to either the *Bacillus coagulans* group (BC group, *n* = 10) or the control group (CT group, *n* = 10) in a ratio of 1:1. Demographic and clinical information including age (years), operative time (min), operation bleeding (mL), diagnosis, and operation types were collected for each patient. Patients in the BC group took *Bacillus coagulans* Tablets, Live (BCTL, TBC169, Qingdao Eastsea Pharmaceutical Co., Ltd., lot No: S202010112), 6 tablets (1.05 × 10^8^ CFU) per dose, while patients in the CT group took 6 placebo tablets (Qingdao Eastsea Pharmaceutical Co., Ltd., lot No: S202107001) per dose. The specific administration time is shown in Figure 1. On the night before laparoscopic surgery, the patients took BCTL (TBC169) for the first time after bowel preparation, with a dosage of 6 tablets (1.05 × 10^8^ CFU). The second dose, also consisting of 6 tablets, was administered 8 h after the completion of anesthesia recovery following the surgery. Subsequently, BCTL (TBC169) was taken 6 tablets every 8 h until the patient experienced intestinal gas expulsion, with the duration of medication typically lasting for 48–72 h.

Each participant submitted a fecal sample after the first postoperative defecation. The primary outcomes of the study were α-diversity and β-diversity, gut microbiota (GM) composition and differential metabolites.

This study was approved by the Ethical Review Committee of Shanxi Bethune Hospital (approval number YXLL-2021-028). All patients were fully informed before they provided informed consents.

### 2.2 Fecal sample collection

Every subject was provided with a fecal kit box (10 mL), and instructed to collect a fecal sample after their first postoperative defecation and then submit it to researchers as soon as possible. Upon submission, the samples were promptly stored at −80°C until high-throughput *16S* rRNA gene sequencing analysis and untargeted metabonomic analysis.

### 2.3 High-throughput *16S* rRNA gene sequencing analysis

Total genomic DNA was extracted from fecal samples using the TGuide S96 Magnetic Soil/Stool DNA Kit (Tiangen Biotech (Beijing) Co., Ltd.). The hypervariable region V3-V4 of the bacterial *16S* rRNA gene were amplified with primer pairs 338F: *5′- ACTCCTACGGGAGGCAGCA-3′* and 806R: *5′- GGACTACHVGGGTWTCTAAT-3′*. PCR products were checked on agarose gel and purified through the Omega DNA purification kit (Omega Inc., Norcross, GA, United States). The purified PCR products were collected and the paired ends (2 × 250 bp) was performed on the Illumina Novaseq 6,000 platform. The qualified sequences with more than 97% similarity thresholds were allocated to one operational taxonomic unit (OTU) using USEARCH (version 10.0). Taxonomy annotation of the OTUs/ASVs was performed based on the Naive Bayes classifier in QIIME2 using the SILVA database (release 138.1) with a confidence threshold of 70%. α was performed to identify the complexity of species diversity of each sample utilizing QIIME2 software. β-diversity calculations were analyzed by principal coordinate analysis (PCoA) to assess the diversity in samples for species complexity. Analysis of variance (ANOVA) was used to compare bacterial abundance and diversity. Linear discriminant analysis (LDA) coupled with effect size (LEfSe) was applied to evaluate the differentially abundant taxa.

### 2.4 Untargeted metabolomics analysis

The details of chemical reagents used in this study are listed in Table 1.

**Table 1:** The details of chemical reagents.

Name	CAS	Purity	Brand
Methanol	67-56-1	LC–MS grade	Merck
Acetonitrile	75-05-8	LC–MS grade	Merck
2-Chloro-L-phenylalanine	103616-89-3	≥98%	Shanghai Aladdin
Formic acid	64-18-6	LC–MS grade	TCI

#### 2.4.1 Sample preparation

Firstly, we weighed 50 mg of freeze-dried fecal sample and added 1,000 μL extraction solution (volume ratio of methanol, acetonitrile and water was 2:2:1, containing internal standard, 2-Chloro-L-phenylalanine, which concentration was 20 mg/L) into the sample. The mixture was vortexed 30 s, and then centrifugated at 12,000 rpm for 15 min at 4°C following 10 min ultrasonic processing. After the centrifugation, we took 500 μL of supernatant for vacuum drying treatment, and then added 160 μL extraction solution (volume ratio of acetonitrile and water was 1:1) to the dried metabolite to make it dissolve again. The re-dissolved sample was vortexed 30 s, processed 10 min by ultrasonic, and centrifugated at 12,000 rpm for 15 min at 4°C. And then we obtained the supernatant for the further metabolomics analysis. Moreover, we took 10 μL supernatant from each sample and mixed together as a quality control sample.

#### 2.4.2 LC/MS analysis

The LC/MS system for metabolomics analysis is composed of Waters Acquity I-Class PLUS ultra-high performance liquid tandem Waters Xevo G2-XS QTof high resolution mass spectrometer. The column used is purchased from Waters Acquity UPLC HSS T3 column (1.8um 2.1*100 mm). The mobile phases in this study were consisted of 0.1% formic acid aqueous solution (solvent A) and 0.1% formic acid acetonitrile (solvent B). The injection volume was 1 μL and the flow rate was 0.40 mL/min. And the gradient system was optimized as follows: 0 ~ 0.25 min, 98% A and 2% B, 0.25 ~ 10 min, 98% A and 2% B, 10 ~ 13 min, 2% A and 98% B, 13 ~ 13.1 min, 2% A and 98% B, 13.1 ~ 15 min, 98% A and 2% B, 15 ~ 16 min, 98% A and 2% B. Waters Xevo G2-XS QTOF high resolution mass spectrometer can collect primary and secondary mass spectrometry data in MSe mode under the control of the acquisition software (MassLynx V4.2, Waters). In each data acquisition cycle, dual-channel data acquisition can be performed on both low collision energy and high collision energy at the same time. The low collision energy is 2 V, the high collision energy range is 10 ~ 40 V, and the scanning frequency is 0.2 s for a mass spectrum. The parameters of the ESI ion source are as follows: Capillary voltage: 2,000 V (positive ion mode) or − 1,500 V (negative ion mode); cone voltage: 30 V; ion source temperature: 150°C; desolvent gas temperature 500°C; backflush gas flow rate: 50 L/h; Desolventizing gas flow rate: 800 L/h.

#### 2.4.3 Data processing and analysis

The raw data collected through MassLynx V4.2 were processed by Progenesis QI software for peak extraction, peak alignment and other data processing operations, and identification was based on the Progenesis QI software and METLIN database. After normalizing the original peak area information with the total peak area, the follow-up analysis was performed. Principal component analysis (PCA) was used to show the overall metabolic differences between groups. According to the grouping information, T test was used to calculate the *p* value of each compound, and the fold change (FC) was calculated. The screening criteria of differential metabolites were *p* value <0.05 and FC > 1.2 or FC < 0.8. MetPA website^1^ was used for pathway analysis.

## 3 Results

### 3.1 Baseline characteristics of patients post-GLs

As described previously, 20 subjects enrolled in this study were randomized into the BC group (*n* = 10) and the CT group (*n* = 10). Baseline characteristics of subjects are shown in Table 2. Eventually, all 20 patients submitted fecal samples successfully. These samples from the BC group and the CT group were labeled as BCD1 ~ BCD10 and CTD1 ~ CTD10, respectively.

**Table 2:** Baseline characteristics of subjects.

		BC (*n* = 10)	CT (*n* = 10)
Age (years)		49.60 ± 7.60	49.40 ± 10.07
Operative time(min)		163.40 ± 47.77	109.40 ± 58.04
Operation bleeding (ml)		39.00 ± 25.14	109.00 ± 181.50
Diagnosis	uterine fibroids	7 (70.0%)	6 (60.0%)
	uterus adenomyosis	2 (20.0%)	1 (10.0%)
	endometrial cancer	1 (10.0%)	3 (30.0%)
Operation types	LTH^a^	9 (90.0%)	9 (90.0%)
	LM^b^	1 (10.0%)	1 (10.0%)

LTH^a^, Laparoscopic Total Hysterectomy; LM^b^, Laparoscopic Myomectomy.

### 3.2 Effect of BCTL on GM composition

#### 3.2.1 α-Diversity and β-diversity

**Figure 2:**
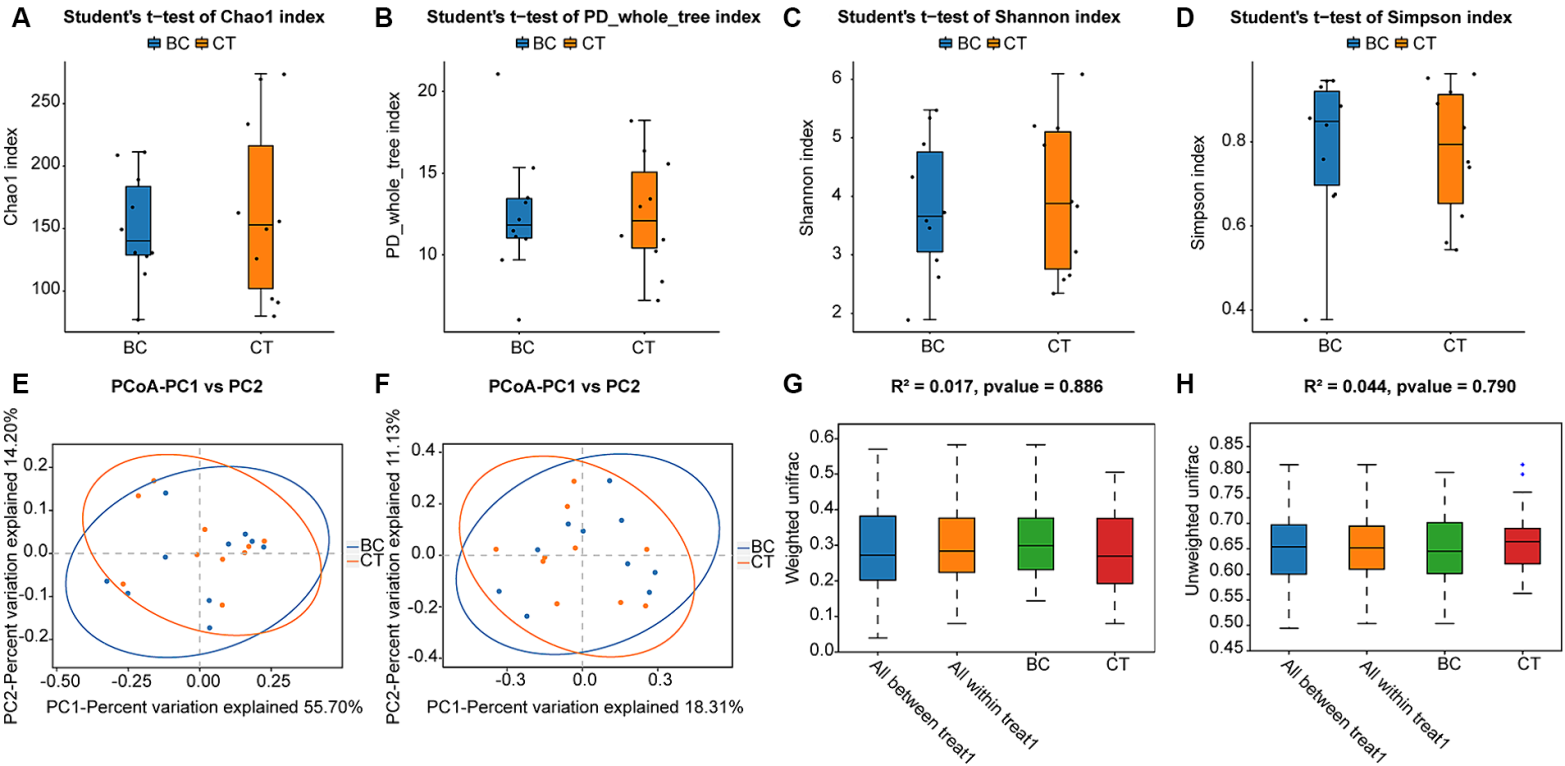
α-diversity data of the BC group and CT group: **(A)** Chao1 index, **(B)** PD whole tree, **(C)** Shannon index, and **(D)** Simpson index. β-diversity data of the BC group and CT group: **(E)** weighted unifrac distances, **(F)** unweighted unifrac distances. The boxplot shows the mean unifrac distances for BC group and CT group: **(G)** weighted unifrac distances (BC vs. CT, *p* = 0.886), **(H)** unweighted unifrac distances (BC vs. CT, *p* = 0.790). (In the boxplot above, “All between” represents the beta-distance data of all inter-group samples, and “All within” represents the beta-distance data of all intra-group samples).

The α-diversity index is used to measure the species richness and diversity within a single sample. In this study, the results of the α-diversity data are presented in Figure 2. Confirmed by student’s t-test, there were no significant differences in α-diversity between BC group and CT group (Chao1, *p* = 0.631; PD whole tree, *p* = 0.998; Shannon, *p* = 0.791; Simpson, *p* = 0.888). As shown in Figure 2C (Shannon index), no significant differences were observed in species evenness.

QIIME was utilized in this study to conduct β-diversity analysis, which enabled the comparison of species diversity among different samples and could be computed based on the weighted unifrac and unweighted unifrac. PCoA and PERMANOVA were used to analyze the β-diversity. No significant difference was observed either between or within groups eventually (Figures 2G,H).

#### 3.2.2 Gut microbiota composition

**Figure 3:**
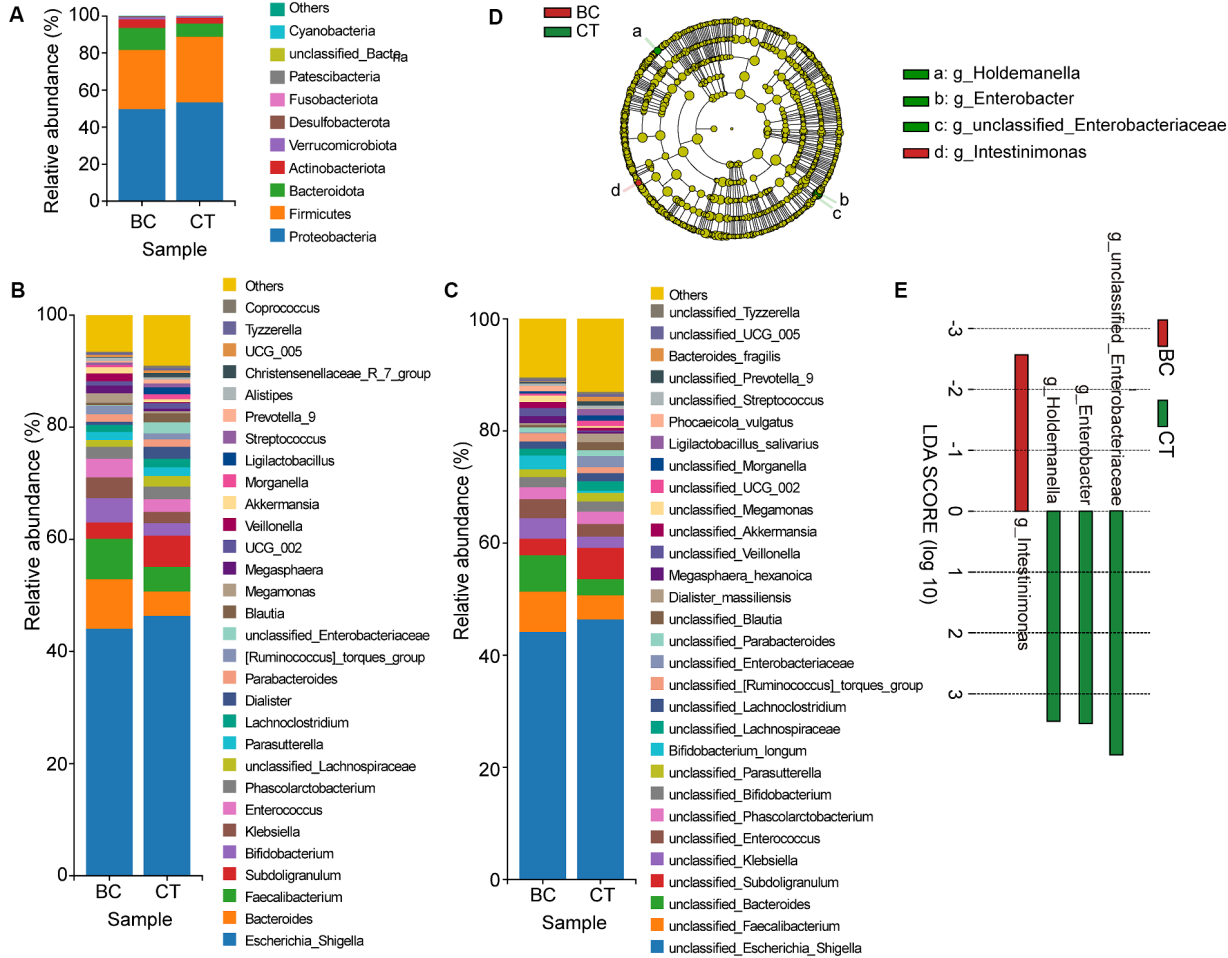
Relative abundance of GM at phylum level **(A)**, genus level **(B)**, and species level **(C)** between two groups; and results of LEfSe **(D)** and LDA scores **(E)**.

Figure 3 displays the relative abundance of the top 10 GM at the phylum level, the top 30 GM at the genus level, and the top 30 GM at the species level between different groups. This visualization provides insight into the composition and distribution of these microbial taxa within the studied groups. The relative abundance of GM was compared using ANOVA, revealing a significant difference in the abundance of *unclassified_Enterobacteriaceae* at the genus level between two groups. Specifically, the CT group exhibited a significantly higher abundance of *unclassified_Enterobacteriaceae* (*p* = 0.043). While there was no difference were observed at the phylum level and the species level.

In the study, LDA coupled with LEfSe were used to identify the biomarkers between BC group and CT group from phylum level to genus level (LDA>2). In CT group, the biomarkers included *Holdemanella*, *Enterobacter* and *unclassified_Enterobacteriaceae* at the genus level. Meanwhile, there were 1 biomarker in BC group, which was *Intestinimonas* at genus level (Figure 3D). The LDA scores of the aforementioned microbiota are shown in the Figure 3E. And the details of biomarkers were shown in Table 3.

**Table 3:** The details of biomarkers.

Biomarker	Abundance	Group	LDA	*p*
*g_unclassified_Enterobacteriaceae*	4.303	CT	4.023	0.004
*g_Holdemanella*	3.225	CT	3.274	0.031
*g_Enterobacter*	3.452	CT	3.703	0.002
*g_Intestinimonas*	2.046	BC	2.566	0.031

### 3.3 Effect of BCTL on metabolic profiles

#### 3.3.1 Variety of fecal metabolic profiles

**Figure 4:**
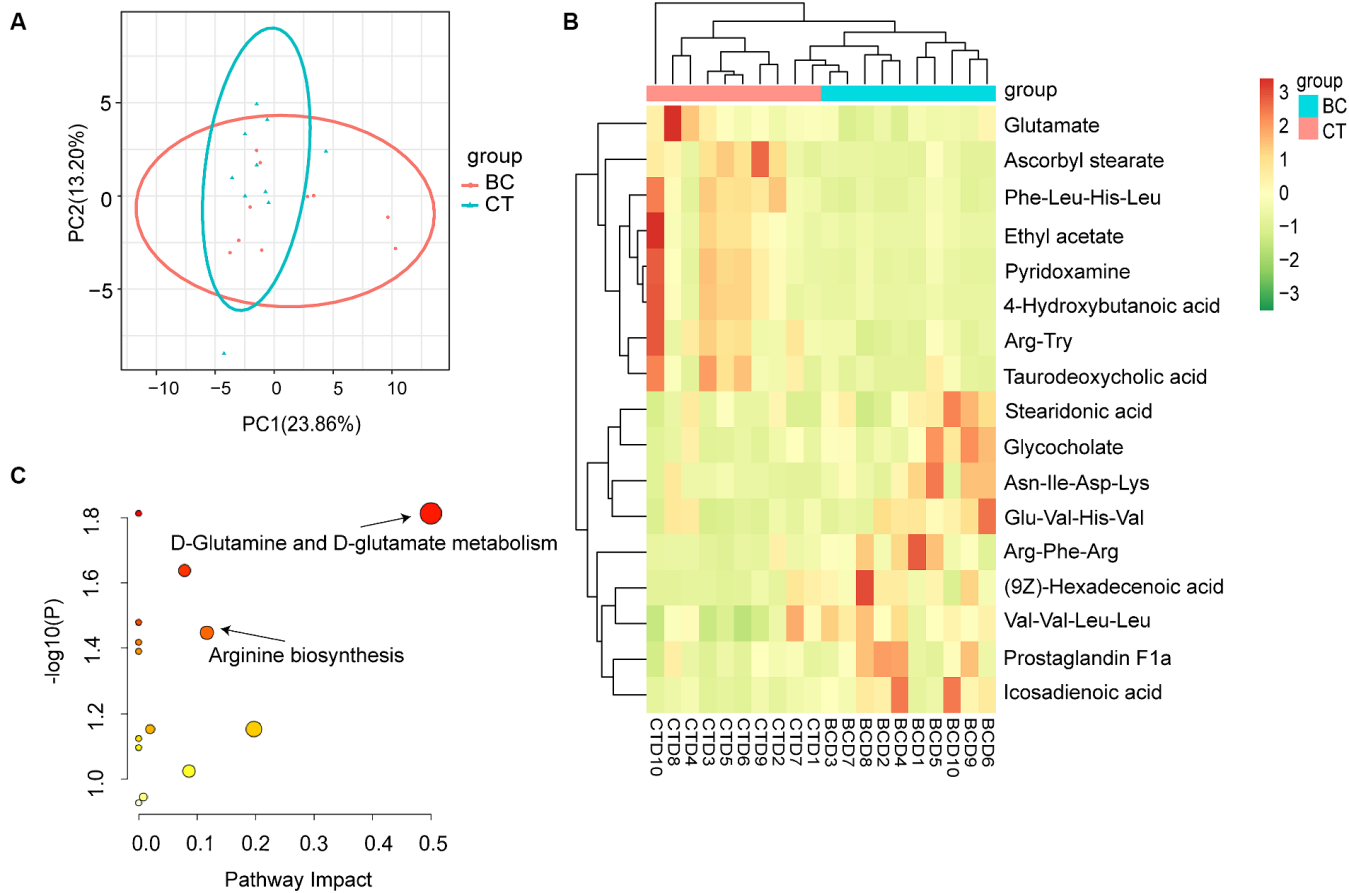
**(A)** PCA score plot. **(B)** Heatmap of the differential metabolites. **(C)** 2 metabolic pathways with significant variations in BC group.

PCA, a multidimensional statistical analysis method with unsupervised pattern recognition, was used to examine overall metabolic differences among samples in each group and the degree of variation among samples within the group. As illustrated in Figure 4A, the scatter plots of the BC and CT groups exhibit partial overlap; however, there is a trend of separation between them.

Human Metabolome Database (HMDB) was utilized to annotate all the identified metabolites, resulting in a total of 70 metabolites being successfully identified. The screening criteria for differential metabolites included *p* < 0.05 and FC > 1.2 or FC < 0.8, and the final differential metabolites can be seen in Table 4. A total of 17 metabolites were considered as differential metabolites, 9 of them were significantly increased, while 8 of them were significantly decreased. The heatmap displaying the 17 differential metabolites is presented in Figure 4B. It allows for a quick observation of the overall trend in metabolite differences between the two groups.

**Table 4:** Statistics of differential metabolites.

Name	FC	*p*	Regulation	Type
Glutamate	0.47	0.032	Down	Amino acid
Taurodeoxycholic acid	0.21	0.022	Down	Bile acid
Glycocholate	4.12	0.021	Up	Bile acid
(9Z)-Hexadecenoic acid	2.20	0.039	Up	Fatty Acyl
Asn-Ile-Asp-Lys	2.87	0.032	Up	Peptide
Arg-Try	0.29	0.007	Down	Peptide
Glu-Val-His-Val	1.93	0.025	Up	Peptide
Ascorbyl stearate	0.12	0.005	Down	Fatty Acyl
Pyridoxamine	0.12	0.008	Down	Pyridines and derivatives
Ethyl acetate	0.10	0.026	Down	Carboxylic acid
Phe-Leu-His-Leu	0.31	0.002	Down	Peptide
4-Hydroxybutanoic acid	0.07	0.009	Down	Fatty Acyl
Prostaglandin F1a	2.03	0.040	Up	Fatty Acyl
Arg-Phe-Arg	2.62	0.020	Up	Peptide
Stearidonic acid	2.02	0.032	Up	Fatty Acyl
Val-Val-Leu-Leu	1.53	0.024	Up	Peptide
Icosadienoic acid	3.46	0.011	Up	Fatty Acyl

#### 3.3.2 Metabolic pathway analysis

The 17 differential metabolites identified in fecal samples have been classified into different categories, as shown in Table 4. These metabolites are categorized as follows: amino acids and peptides (7), bile acids (2), fatty acyls (6), carboxylic acid (1), and pyridines and derivatives (1). These differential metabolites were imported into the MetPA website (see footnote 1) for pathway analysis. The metabolic pathways with the impact >0.1 and *p* < 0.05 were considered potential target pathways. Compared with CT group, there were 2 target metabolic pathways in BC group: D-glutamine and D-glutamate metabolism, and arginine biosynthesis (Figure 4C).

### 3.4 Correlation analysis between differential metabolites and microbiota

Correlation analysis between the differential metabolites and differential microbiota was performed using the Hmisc package in R (version 3.6.1). The screening criteria for correlation were set as *r* > 0.5 or *r* < −0.5, with a significance level of *p* < 0.05. Ultimately, only one significant correlation was observed: *Intestinimonas* showed a positive correlation with Arg-Phe-Arg (*r* = 0.8056, *p* = 1.82 × 10^−5^).

## 4 Discussion

### 4.1 Impact of taking BCTL after GLs on GM composition

In this study, no statistically significant differences were observed in α-diversity and β-diversity between the BC group and CT group. One possible explanation for this finding could be that the intervention duration of BCTL was relatively short.

However, it is worth noting that despite the lack of significant differences in α-diversity and β-diversity between two groups, a notable finding emerged. Specifically, the abundance of *unclassified_Enterobacteriaceae* at the genus level was found to be significantly higher in the CT group compared to the BC group. This suggests a potential association between the administration of BCTL and the decreased abundance of *unclassified_Enterobacteriaceae* in the BC group. *Unclassified_Enterobacteriaceae* belongs to facultative anaerobic pathogens, and it is known to primarily degrade proteins and produce indoles. When the concentration of indoles rises beyond a certain threshold, it can disrupt the balance within the intestine (Verbeke et al., 2014; Seo et al., 2017). In the context of this study, the observed difference in abundance of *unclassified_Enterobacteriaceae* between the BC group and CT group suggests that the probiotic BCTL intervention may have the potential to inhibit the proliferation of harmful *Enterobacteriaceae* and help maintain intestinal balance.

In addition, the biomarkers with statistically significant differences between the two groups were identified using LDA and LEfSe analysis. Apart from *unclassified_Enterobacteriaceae*, the biomarkers specific to the CT group comprised *Holdemanella* and *Enterobacter*. Furthermore, the biomarkers in the BC group was *Intestinimonas*. It is possible that the recovery of intestinal function after GLs could be attributed to the alterations of the above GM.

*Holdemanella* was found to be related to a risk of anxiety and depression (Chen et al., 2021), however it was also considered a potential contributor to the stable state of health (Zhang et al., 2022). These differences provided a challenge of judging whether *Holdemanella* is a beneficial bacterium at present. The genus *Enterobacter* is recognized as a pathogenic bacterium in most cases (Ye et al., 2006), and it was found to be more prevalent in adults with chronic constipation (Khalif et al., 2005), suggesting a potential correlation with impaired intestinal motility. The enrichment of *Enterobacter* in the CT group indicates an imbalance in the intestinal microflora among patients. This imbalance has the potential to disrupt normal intestinal function and motility (Zhang et al., 2018). Therefore these kinds of bacteria might be related to delayed recovery of intestinal function.

As for biomarkers in BC group, the genus *Intestinimonas* was found to be a butyrate- producing bacterium (Kläring et al., 2013). Butyrate has been shown to enhance the barrier function of human colonic epithelial cell line, and influence the level of intestinal inflammation (Bach Knudsen et al., 2018; Nielsen et al., 2018). However, in this study, according to results of metabolomics analysis, the concentration of butyrate was not found to be up-regulated. This discrepancy could potentially be attributed to the relatively short time interval between the initiation of medication and the collection of fecal samples, which did not allow for the capture of changes in butyrate.

### 4.2 Impact of taking BCTL after GLs on metabolic profile

The results of PCA demonstrated a trend of separation of scatter points between the two groups. This suggests that there are discernible differences in metabolic profiles between patients who were administered BCTL and those who were given placebos. The observed separation supports the notion that the treatment with BCTL has an impact on the metabolic profile within the study population.

#### 4.2.1 Variations in metabolic pathways

Glutamic acid (Glu) plays a significant role in maintaining intestinal mucosal barrier function. It responds to oxidative stress by promoting cell growth and preserving membrane integrity (Jiao et al., 2015). The occurrence and development of inflammation caused by oxidative stress are important factors in disrupting the balance of gut microbiota (Wang et al., 2023). In this study, we speculated that the down-regulation of Glu in patients taking BCTL may be attributed to increased absorption by intestinal epithelial cells. BCTL treatment might enhance the absorption and utilization of Glu in the intestine, contributing to the maintenance of intestinal balance and accelerated recovery of intestinal function following GLs. According to previous studies, there was a correlation between arginine consumption and inflammation in colon (Monaghan et al., 2023). In the study, BCTL might protect against intestinal inflammation through the down-regulation of Arg-Try, and the up-regulation of Arg-Phe-Arg. Additionally, there was a positive correlation between *Intestinimonas*, an anti-inflammatory microorganism, and Arg-Phe-Arg. This indicates that the increase in the latter’s level may be attributed to the influence of the former. The levels of *Intestinimonas* and Arg-Phe-Arg were both increased, which has a positive effect on the recovery of clinical intestinal function.

#### 4.2.2 Metabolites related to the immune system

According to previous research, in rodents, the degree of insufficient gastrointestinal motility or intestinal obstruction was proportional to the degree of intestinal handling and inflammation; and the down-regulation of inflammation in the colon was associated with promoting colon contraction and reducing intestinal transit time (Kalff et al., 1998; Wang et al., 2023). In humans, although minimally invasive surgery such as laparoscopy has limited handling of the intestine, it still causes transient inflammation and delays the recovery of gastrointestinal motility (The et al., 2008). And there was evidence of a causal relationship between intestinal mucosal inflammation and changes in intestinal sensory and motor function in humans (Collins, 1996). The following differential metabolites related to the immune system may have a potential association with the accelerated recovery of intestinal motility in patients receiving BCTL.

Taurodeoxycholic acid (TDCA), a secondary bile acid, its enrichment could drive the inflammatory immune response in the small intestines of mice; and patients with inflammatory bowel disease had significantly higher levels of TDCA compared to normal individuals (Liu et al., 2023). This suggests a relationship between TDCA levels and intestinal inflammation. The observed down-regulation of TDCA in BC group may due to the reduction of intestinal inflammation.

(9Z)-Hexadecenoic acid, also known as palmitoleic acid (POA), is increasingly recognized as a health biomarker with important physiological and pathophysiological functions (Bermúdez et al., 2022). Previous studies conducted on animal models or cell cultures have shown that POA exhibited significant anti-inflammatory properties (Astudillo et al., 2018; de Souza et al., 2018). But its role in humans has not yet been fully explored. A study demonstrated the potential benefits of POA supplementation for patients with inflammatory bowel disease (Bueno-Hernández et al., 2017), suggesting a possible negative correlation between POA levels and intestinal inflammation. In this study, the up-regulation of POA in the BC group indicated a potential relationship between BCTL and the inhibition of intestinal inflammation.

Ascorbyl stearate (Asc-s) had shown anti-tumor properties without causing damage to normal cells, and due to its lipophilicity, it was easy to penetrate the cell membrane and be absorbed by cells (Fang et al., 2006; Mane and Kamatham, 2019). Subjects in this study included patients with gynecological tumors, and the down-regulation of Asc-s in BC group may be related to its uptake by cells, which suggests that BCTL may had an influence on promoting the uptake of anti-tumor active factors in patients with tumors.

Pyridoxamine is a beneficial anti-glycation agent. It can mitigate the detrimental effects of saccharification process caused by fermentable carbohydrates in the intestine of mice, reduce the mucosal irritation, and maintain the balance of intestinal mucus barrier (Kamphuis et al., 2022). Probiotic supplementation was found to increase pyridoxamine level in mouse fecal samples (Huang et al., 2021). However, in this study, it was down-regulated in BC group. This may be because the factors affecting its concentration in humans are complicated, including ingestion and vitamin B6 metabolism.

Stearidonic acid (SDA) belongs to long-chain omega-3 polyunsaturated fatty acid, and has been recognized for its potential health benefits (Li et al., 2022). It had been proven to possess anti-inflammatory and immunity-enhancing properties (Hsueh et al., 2011; Lucchinetti et al., 2022). As well as icosadienoic acid, which is an omega-6 fatty acid, might play a role in modulating inflammatory responses (Sergeant et al., 2016). The absence of these anti-inflammatory fatty acids may be associated with an intestinal inflammatory response (Galler et al., 2022). In this study, the up-regulation of SDA and icosadienoic acid in the BC group suggests their potential involvement in intestinal anti-inflammatory activity.

4-Hydroxybutyric acid (also known as gamma-hydroxybutyrate or GHB) is a precursor and a metabolite of gamma-aminobutyric acid (GABA) (LeBeau et al., 2006). GABA is one of the main neurotransmitters and plays a role in the gut-brain axis communication (Kasarello et al., 2023). GABA can reduce oxidative stress in the small intestine of mice, thereby alleviating intestinal barrier damage (Ji et al., 2023). In this study, BCTL may have an impact on GABA levels, which in turn influences the variation in 4-hydroxybutyric acid (GHB).

#### 4.2.3 Metabolites related to intestinal motility

It was reported that Glycocholate (GC) can chelate calcium. Ca^2+^ is a signal involved in motility, can bind to glycine-conjugated bile salt micelles (Rajagopalan and Lindenbaum, 1982), thus increasing the intake of Ca^2+^ in the intestine. The enrichment of GC observed in this study may indicate an increase in intestinal Ca^2+^ concentration, which could be associated with improved intestinal motility.

It is well-recognized that potassium channel is important in gastrointestinal system regulation. According to an animal research, ethyl acetate (ETAC) suppressed both spontaneous and K^+^-induced contractions of rabbit jejunum. The inhibition of ETAC on transit of contents in the small intestine was similar to atropine sulfate, which is a drug with strong anticholinergic activity on intestinal transit (Rouf et al., 2003; Qazi et al., 2022). In conclusion, ETAC showed a negative correlation with intestinal motility. Therefore the down-regulation of ETAC in BC group suggests an increase in intestinal motility in patients taking BCTL.

Prostaglandin F1a (PGF1a) can lead to the contraction of circular muscle of the intestine (Wilson, 1974). And in an animal study, intra-arterial infusion of PGF could obviously stimulate intestinal motility (Shehadeh et al., 1969). The increase of PGF1a in BC group was related to the accelerated recovery of intestinal motility.

In this study, the researchers investigated the potential mechanism of TBC169 in promoting intestinal function recovery in patients undergoing GLs from the perspective of gut microbiota and metabolites. The degree of impact of gynecological diseases on the intestines of patients, as well as the amount of bleeding during gynecological laparoscopic surgery, might be confounding factors in the study. The greater the degree of intestinal involvement and the greater the amount of bleeding during surgery, the less effective BCTL will be in restoring intestinal function. To address the first confounding factor, we had taken measures to control its interference by selecting similar disease types and laparoscopic surgical techniques. However, in this study, the second confounding factor had not been controlled, and it is worth noting that the amount of bleeding in the CT group was significantly higher than that in the BC group. Moving forward, we plan to establish strict inclusion criteria for assessing the amount of bleeding in patients to address this issue in future studies. In addition, there were several limitations in this study. First of all, we found a substantial variation in the patient dosage across different clinical studies involving *Bacillus coagulans.* After reviewing the literature, in this study, we slightly up-regulated the dosages of *Bacillus coagulans* administered to patients compared to the instructions. Future studies should further investigate the optimal dosage of *Bacillus coagulans* for patients to accelerate the recovery of intestinal function. Secondly, the sample size in this study was relatively small. And we hope that we could expand the sample size for future research. Furthermore, due to the exploratory nature of this study, a double-blind method was employed exclusively for the patients, while the doctors and nurses were not involved in blinding procedures. In future research, we will add a preoperative sample group to collect fecal samples from patients before laparoscopic surgery. Then compare this group with the postoperative placebo group and the postoperative medication group to explore the changes in metabolites and microbiota in feces before and after surgery, and verify the changes in microbiota and metabolic profile before and after taking BCTL. We hope to provide more and stronger evidence for BCTL to promote postoperative intestinal function recovery in clinical laparoscopic surgery in the future.

## 5 Conclusion

In conclusion, this study demonstrated that consuming *Bacillus coagulans* TBC169 after gynecological laparoscopic surgery might inhibit the proliferation of harmful *Enterobacteriaceae*; mainly influence 2 pathways including D-glutamine and D-glutamate metabolism, and arginine biosynthesis; and regulate metabolites related to immunity and intestinal motility; which can help regulate immune function, maintain intestinal balance, promote intestinal peristalsis, and ultimately accelerate the recovery of intestinal function.

## Data availability statement

The datasets presented in this study can be found in online repositories. The names of the repository/repositories and accession number(s) can be found at: 16S rRNA Gene Sequencing Data: NCBI – Accession Number: [PRJNA1041567], LC-MS Data: Accession Number: [doi: https://doi.org/10.5061/dryad.4mw6m90h9].

## Ethics statement

The studies involving humans were approved by the Ethical Review Committee of Shanxi Bethune Hospital. The studies were conducted in accordance with the local legislation and institutional requirements. The participants provided their written informed consent to participate in this study. Written informed consent was obtained from the individual (s) for the publication of any potentially identifiable images or data included in this article.

## Author contributions

WG: Writing – review & editing. YY: Writing – original draft. ZG: Writing – original draft. JZ: Writing – review & editing. WC: Supervision, Writing – review & editing.

## Footnotes

^1^ https://www.metaboanalyst.ca/
